# 

**Published:** 1888-03-24

**Authors:** 


CONTENTS.

**Makers or Labellers** - - - 421

**Words of Consolation.** Chapter LXX.—The Sufferings of Christ - - - 422

**The Doctor's Pulpit.**—The Bees and the Hive - - - 423

**Round about the Asylums.** III.—By a MEDICAL SUPERINTENDENT - - - 424

**Aids to Nursing.** By M. M. BASIL, M.A., M.B., Author of "The Commoner Accidents to Life and Limb."—Food and Feeding - - - 425

**Scotch Physicians and Surgeons.** By C. G. FURLEY.—Sir Robert Christison - - - 426

**Incompleteness** - - - 427

**Notes and News** - - - 428

**Everybody's Page** - - - 430

**Annotations.**—Bone Setting in the Black Country.—The *Lancet* and the College of Physicians.—Emigration and Self-Help - 431

**Rachel Challice's Vow.** By LINA NEVILL, Author of "A Future on Trust," etc. - - - 432

**The Nursing Mirror.**—The Nice Nursing Home.—More about Nice.—The British Nurses' Association.—Free Training in Midwifery, etc. - - - 434

**The Editor's Letter-Box.**—Workmen's Contribution to Leeds Infirmary.—Prayers in Hospitals, etc. - - - 435

**Amusements with Profit for all Ages.**—For Men and Women.—Children's Corner.—Puzzles, etc. - - - 436

